# Enhancer RNAs: similarities with both lncRNAs and mRNAs reveal novel functions

**DOI:** 10.1080/15476286.2026.2682066

**Published:** 2026-06-09

**Authors:** Pavel A. Vlasov, James L. Manley

**Affiliations:** Dept. of Biological Sciences, Columbia University, New York, NY, USA

**Keywords:** Enhancers, translation, RNA, eRNA, lncRNA, RNA processing

## Abstract

Cells produce numerous types of RNAs. Among these, transcripts produced by RNA polymerase II include protein-coding mRNAs as well as a variety of long noncoding RNAs. In this latter group, enhancer (e) RNAs constitute a class of RNAs transcribed from enhancer sites. Although eRNAs are typically unstable and degraded rapidly, multiple roles related to enhancer function have been suggested. But eRNAs also share similarities with mRNAs, such as in a limited number the presence of translated open reading frames. Indeed, other “noncoding” RNAs have also been found to contain coding sequences, and together these transcripts blur the line between coding and noncoding. Here, we review current models of eRNA function, the discoveries that led to them, and additional functions, specifically the potential for translation. We also review the characteristics of proteins encoded by such “noncoding” transcripts, and their possible implications regarding the function and evolution of both eRNAs and mRNAs.

## Introduction

Enhancer RNAs (eRNAs) constitute a class of long noncoding RNAs (lncRNAs) transcribed by RNA polymerase II (RNAPII) from enhancer sites as part of the process of enhancer activation and regulation of target genes. Despite their status as distal regulatory regions, enhancers require transcription factors and RNAPII as part of their activation and function, and frequently display sequence similarities to promoters [1,2]. An important function of eRNAs involves interaction with the Mediator complex [3] as part of the process of enhancer looping, a function of enhancers identified before eRNAs were discovered [4,5]. Other functions have also been proposed, including for example a role in the formation and function of nuclear condensates containing the transcriptional machinery [6,7]. While exceptions exist, eRNAs are typically nuclear and unstable, and their accumulation is regulated by multiple layers of RNA modification and quality control, including targeting by NEXT and PAXT complexes for turnover by the nuclear exosome [8,9] and early transcription termination by the RNAPII-associated Integrator complex [10].

Despite being considered ‘noncoding’, a growing number of lncRNAs have been found to contain open reading frames (ORFs) that can be translated to produce peptides or even proteins with a variety of functions, from transcriptional regulation to cellular stress responses (e.g. [11,12]), in some case with implications for diseases including cancer (reviewed by [13,14]). Ribosome profiling and proteomic studies have revealed translation events occurring on many canonically noncoding RNA sequences [15,16], and eRNAs are a promising source for such translation events, given their similarities with mRNAs in transcription and processing. Recent results have in fact identified such translation events in a subset of eRNA transcripts, capable of producing stable proteins as large as ~45 KDa [17]. While ribosome profiling and mass spectrometry (MS) have limitations, such as the former not differentiating functional vs non-functional translation and the latter being less reliable for detecting lower-abundance proteins, results with these methods have identified many novel peptides and proteins.

ORF-containing lncRNAs, including eRNAs, are also a potential source of novel genes, arising through the process of de novo gene birth (e.g. [18]). Enhancers have a higher mutation rate than promoter regions, associated with their status as CpG island-rich sequences [19–21], and computational genomics studies have previously identified species-specific ORFs associated with enhancer sites [22]. Furthermore, functional human-specific lncRNAs and lncRNA-encoded peptides have previously been identified (e.g. [23]), while another computational study predicted families of de novo proteins in *Drosophila* with possible functions in nuclear condensates [24].

In this review, we discuss the main discoveries leading to our current understanding of eRNAs and their functions and findings from multiple areas of RNA, genomics and proteomics research that have revealed novel eRNA functions. These include notably the presence in some of active ORFs and the possibility that such ‘hidden’ ORFs, in lncRNAs as well as in eRNAs, may serve as a reservoir for de novo gene birth. The interested reader is referred to several reviews on eRNA and other lncRNA function and regulation [25–29], on possible roles of eRNAs in disease [30–32], as well as to reviews of de novo gene birth [18,33,34].

## RNA polymerase II and lncRNAs

RNAPII is historically associated with transcription of protein-coding genes to generate mRNAs. However, synthesis of mRNAs covers only a subset of the functions of both RNAPII and its RNA products. For example, lncRNAs constitute a large group of transcripts, now realized to be at least as abundant in number as mRNAs [35]. These include, for example, X-inactive specific transcript (Xist), perhaps the first lncRNA discovered, over 30 years ago [36–38]. Subsequent findings in transcriptomics following the mapping of the human genome revealed an extensive landscape of transcription beyond the known protein-coding genes [39], leading to projects such as ENCODE, which detected transcription of nearly 75% of the human genome [35,40,41].

lncRNAs are generally defined as noncoding RNAs over 200 nucleotides (nts) in length and are typically capped, polyadenylated and often spliced, similar to mRNAs. These transcripts perform a wide variety of roles in the cell and can be nuclear or cytoplasmic. The aforementioned Xist is involved in X-chromosome inactivation, functioning in part by recruiting the SPEN protein, which recruits SMRT, a component of a transcriptional corepressor complex, that in turn activates histone deacetylases leading to the formation of an inactive Barr body [42]. Similarly, other lncRNAs are often also involved in chromatin modification as part of transcriptional control. For example, HOTAIR, as part of a larger lncRNA family, interacts with polycomb repressive complex 2 and other chromatin-modifying proteins, involved in repression [43,44] or activation [45]. lncRNA roles are not restricted to epigenetic modification. One such example, NEAT1, is necessary for the maintenance of nuclear architecture, specifically the formation of paraspeckles [46], while more recent studies have also identified it as a component in complexes required for maintaining genomic integrity [47].

Given their repertoire of regulatory roles, many lncRNAs are involved in stem cell function and differentiation [48–50], including many whose loss results in developmental defects across a variety of systems [51]. In addition to the previously mentioned chromatin and nuclear architecture roles, other lncRNAs discovered in stem cells are involved in processes such as splicing regulation [52], mRNA stability and turnover [53] and translational repression [54]. For example, multiple lncRNAs have been found to regulate WNT signalling through RNA-protein interactions stabilizing pathway member proteins [55,56]. Recent work utilizing CRISPRi screens in stem cells has identified hundreds of growth-affecting lncRNAs, with examples of both positive and negative effects, including RNAs regulating well-known factors such as OCT4 and VRTN [57]. As with many other genes involved in stem cell function, lncRNAs have also been linked to cancer. These include for example lncRNAs involved in p53 activation [58], DNA repair pathways [59] and promotion of metastasis [60–62]. These properties have made lncRNAs novel targets for cancer diagnosis and treatment.

## RNA surveillance and lncRNA regulation

In contrast to the functional lncRNAs described above, other lncRNAs are unstable and subject to rapid turnover in the nucleus as part of normal cellular function. This turnover is an important part of RNA quality control, in which non-functional RNAs from cryptic transcription, premature termination or other errors are degraded by the nuclear exosome complex [63–65]. This is part of a wider regulatory network within the nucleus that determines which RNAs accumulate and are exported to the cytoplasm for translation [66], and prevents deleterious RNA accumulation, which can negatively impact various aspects of cellular function [67,68]. This surveillance also extends to events during transcription, with multiple complexes terminating non-productive pervasive transcription events. For example, the Integrator, which will be discussed in more detail later, can terminate transcription early on [69,70]. SiRNA-mediated depletion of exosome subunits or associated factors results in upregulation of many different transcripts, including products of bi-directional transcription from promoter regions, known as PROMPTs (‘promoter upstream transcripts’) or upstream antisense (ua) RNAs [71–73].

Several protein complexes are involved in targeting RNAs to the exosome for degradation, with different complexes interacting with different target RNA species. For example, the nuclear exosome targeting complex (NEXT) is responsible for the previously described uaRNA/PROMPT surveillance, along with other targets such as 3’ extended small nuclear RNAs [74–77]. Another complex, poly(A) tail exosome targeting complex (PAXT)/polysome protector complex (PPC) also targets polyadenylated uaRNAs/PROMPTs, prematurely terminated RNAs (ptRNAs) as well as eRNAs, which are typically longer than NEXT targets [8,78,79].

Multiple RNA surveillance complexes, including NEXT and PAXT/PPC, contain the RNA helicase Mtr4. Mtr4 functions by interacting directly with the exosome and unwinding RNA for exosome digestion [29,80]. Mtr4 alone is capable of interacting with and unwinding RNA, albeit more slowly than in a complex, and cryo-electron microscopy has shown Mtr4 helping to guide the unwound RNA to the exoribonuclease site of the exosome [81]. Notably, NEXT and PAXT/PPC bind Mtr4 in a mutually exclusive manner. Mtr4 connects the exosome to either the PAXT/PPC complex for polyadenylated transcripts or NEXT for non-polyadenylated RNAs [8], with evidence for different ‘classes’ of transcription start sites producing targets for both exosome-targeting complexes [82]. Together, these complexes allow many different RNA types, including eRNAs, as we discuss more below, to be targeted for exosome-mediated turnover, while also providing a layer of redundancy for transcripts that escape NEXT [29,83].

The above interactions can also involve additional RNA surveillance complexes. For example, the cap binding complex is associated with distinct proteins to determine the fate of multiple RNAs [84]. This includes interaction with the highly conserved protein ARS2 and the NEXT complex to stimulate exosomal degradation of typical NEXT targets such as extended forms of short RNAs produced by transcription termination readthrough [85,86]. Mtr4 is also involved in the competition between RNA degradation factors and RNA export factors such as ALYREF [87,88]. Meanwhile, NRDE2, a protein involved in splicing and RNA interference, was found to bind Mtr4 directly to inhibit its recruitment to the exosome [89], while a separate complex containing NRDE2 and Mtr4 was found to function in DNA repair [90].

Mtr4 depletion leads to increased stability and accumulation of NEXT and PAXT/PPC targets. In the case of PAXT/PPC, this ultimately results in their export to the cytoplasm and in some cases interaction with ribosomes [79,88]. All of this points to a wider role than originally thought for Mtr4 in determining RNA fates, for either degradation or export, and as a key component in complexes that function in a range of RNA, and even DNA, surveillance processes. The molecular mechanisms of RNA quality control maintain the precise balance of RNA transcription, modification and degradation required for normal cellular function, and Mtr4 appears to be central to this network [25,28,91]. Notably, Mtr4 levels are not constant across cell types, varying between different tissues, development stages and normal vs cancer states [92–94], pointing to a possible role for the helicase, and by extension RNA stabilization, in regulating cell fate.

## Enhancers, eRNAs, and their functions

Enhancers are promoter-distal regulatory elements that play critical roles in gene activation. They are characterized by specific histone modification patterns, typically displaying histone H3 lysine 4 monomethylation and lysine 27 acetylation, and are capable of activating transcription of genes kilobases away [95,96]. Both enhancers and promoters are bound by multiple transcription factors, chromatin remodellers such as p300, and RNAPII [97,98], and both undergo transcriptional pause-release by DSIF and P-TEFb [99–101]. The bi-directional nature of eRNA transcription, which is also frequently observed at promoters [73], is a key feature of active enhancers [98,101]

**Figure 1.**
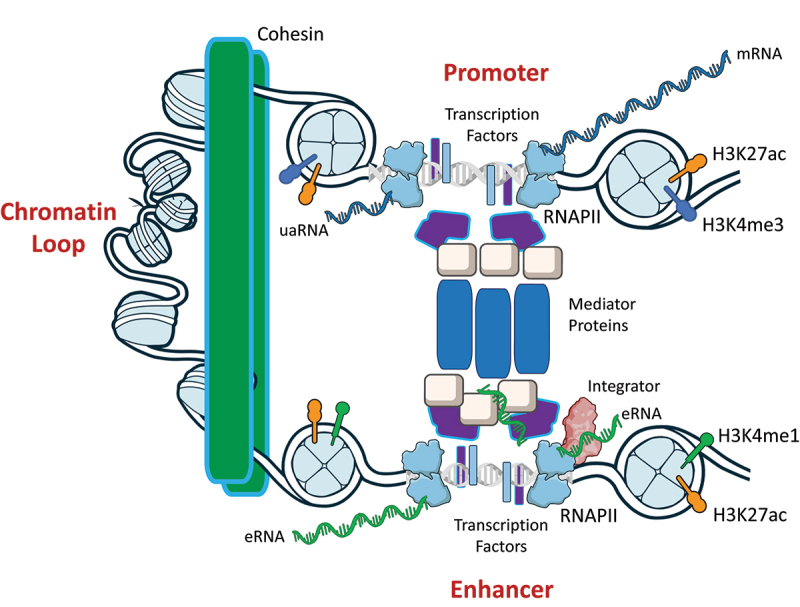
Enhancer loop structure, function and eRNA involvement.

Several models have been put forth to explain the enhancer-mediated promoter activation [102–105]. While evidence has emerged that there is unlikely to be a single mechanism, considerable support exists for the idea that enhancers and promoters interact through the process of chromosome looping, where these elements are brought into close physical proximity [104,106]. Two major protein complexes involved in this process are Mediator and Cohesin, which were found early on to be associated with enhancer and promoter regions connected by chromatin loops [107,108], see Figure 1. Mediator is a large and conformationally flexible complex involved in multiple transcriptional activation and regulatory events [3]. Cohesin is a ring-like complex involved in chromatin looping via the extrusion of chromatin through its ring structure [109–111], a process requiring transcription factors bound to both the enhancer and promoter [112].

Enhancer looping is a prominent model for enhancer function, linking enhancer and promoter sites for transcriptional activation. Enhancer sites are marked by histone 3 lysine 4 monomethylation (H3K4me1), while promoters are marked by histone 3 lysine 4 trimethylation (H3K4me3), and both sites are modified by acetylation at histone 3 lysine 27 (H3K27ac) when active. During enhancer activation, transcription factors and RNA polymerase II (RNAPII) bind to the enhancer site, leading to eRNA transcription. eRNAs that are cleaved by the Integrator complex can then function in the assembly of the Mediator complex. The Mediator links the active enhancer to the target promoter, while the Cohesin complex forms a ring around the chromatin to form the loop. These interactions bring the enhancer and promoter region into closer physical proximity, allowing the enhancer to enhance transcription factor and RNAPII binding to the promoter, leading to mRNA, and in many cases upstream antisense (ua) RNA, transcription.

Enhancers can also exist as larger regulatory regions known as super-enhancers (SEs). SEs can be 10 KB or more in size, contain large numbers of transcription factors bound across multiple sites [98], and regulate multiple target genes [113–115]. SEs have also been found to play major roles in regulation of genes associated with cell fate determination [116–120]. Notably, eRNAs transcribed at SEs have also been implicated in similar regulatory events associated with development and disease [121–124], and as we discuss in the following paragraphs, in some cases may act directly in enhancer function.

As mentioned above, eRNAs are a type of lncRNA. These transcripts are typically unstable, localized in the nucleus, 5’ capped but not spliced, and include both polyadenylated and non-polyadenylated species [27,125–129]. As the documented number of enhancers has increased, so has the number of eRNAs. Indeed, there are now thought to be a remarkable 300,000 or more, with activity detected across a wide range of cell/tissue types [130,131]. RNAPII association with and transcription of enhancers is part of a wider similarity between enhancer and promoter sites [1,99], including the ability of promoters to act as enhancers of other distal promoters [2]. Alongside the discovery of enhancer transcription, lncRNAs associated with enhancers that were capable of activating transcription from nearby promoters in an enhancer-like manner were described [4,132]. These two mechanisms were later found to be part of the same system, as eRNAs play an important role in the enhancer function [133,134].

Many eRNAs have been found to function directly in enhancer activation. For example, early studies of eRNA-like transcripts found that they associate directly with Mediator and that this association is necessary for enhancer-promoter interactions and subsequent activation of target gene transcription [5]. This study, which has been extended by an additional work [3,135], also identified disease-causing mutations in the Mediator subunit MED12 that are associated with decreased eRNA interaction. Multiple studies have also found that certain eRNAs are involved in enhancer loop formation, functioning in cis to enhance specific enhancer-promoter interactions for their respective target genes, and are required for subsequent transcription factor binding and transcription activation [96,135–137]. Furthermore, based on in vitro studies, the enhancer-promoter interaction appears to be mutual, with promoter activity increasing transcriptional output at the enhancer as well as vice versa [138].

In addition to chromosome looping, current models for enhancer-promoter interactions often involve the formation of transcriptional condensates. Such condensates are membrane-less organelles formed from proteins (including RNAPII and Mediator) and nucleic acids at active transcription sites by liquid-liquid phase separation (LLPS) [6,102,139,140] and are part of a wider family of nuclear condensates suggested to function in many processes from DNA repair to RNA processing [7]. Transcriptional condensate formation appears to be stimulated by the transcription of short RNAs (eRNAs) and arrested by bursts of longer (mRNA) RNA transcription [141,142], and perturbations of eRNA processing can disrupt this process [143]. This transcriptional condensate model has also been proposed as the mechanism behind transcriptional bursting events at SE sites [144,145].

eRNAs can also regulate gene expression by driving epigenetic changes. This occurs through the recruitment of chromatin remodelling [146] and histone modification [96] complexes, the latter of which bring about H3K27 acetylation at both promoter and enhancer sites. These changes lead to transcriptional activation through increased chromatin accessibility and are lost by depletion of either the protein complexes or eRNAs involved in the process [147]. Furthermore, in the case of aberrant enhancer activation in cancers such as leukaemia, eRNAs have been identified in association with high-density condensates associated with transcription factor binding, chromatin accessibility and Mediator interaction [148,149], further supporting eRNA function in these processes.

The polyadenylation status of eRNAs remains somewhat ambiguous. Notably, this sort of ambiguity may not be exclusive to eRNAs, as recent long-read sequencing of 5’ capped RNAs has identified both polyA+ and polyA- variants of many transcripts, often associated with different lengths and shifted transcription start sites [125]. Some complexes involved in eRNA turnover, such as PAXT/PPC, typically target polyA+ lncRNAs, and depletion of Mtr4 or other PAXT components decreases this function [79,88,150]. However, eRNA expression and function are also dependent on the Integrator complex, which typically produces polyA- transcripts [10,151]. The Integrator associates with RNAPII [152,153], and one of its primary roles is the termination of non-productive transcription events through endonucleolytic cleavage of the RNA transcript, releasing the stalled RNAPII for productive transcription events [151,154,155]. eRNA termination by the Integrator appears to be necessary for enhancer function, as Integrator depletion leads to decreased enhancer-promoter interaction resulting in decreased transcription of target genes [10,154]. However, early evidence for widespread enhancer transcription detected eRNAs in both polyA+ and polyA- datasets [35]. Studies investigating individual eRNAs also identified both polyA+ and polyA- versions of the same transcripts [135], and both NEXT and PAXT interactions have been identified with transcripts from the same enhancer [8]. Furthermore, long-read sequencing of 5’ capped RNAs has confirmed the presence of both polyA+ and polyA- transcripts from enhancer sites [125]. Notably, a global analysis in erythroid cells found that SEs produce higher levels of polyA+ eRNAs compared to typical enhancers [121], and it has also been observed that lncRNAs associated with SEs, likely eRNAs or perhaps evolved from eRNAs, are more likely to contain introns and undergo splicing [156–158].

**Figure 2.**
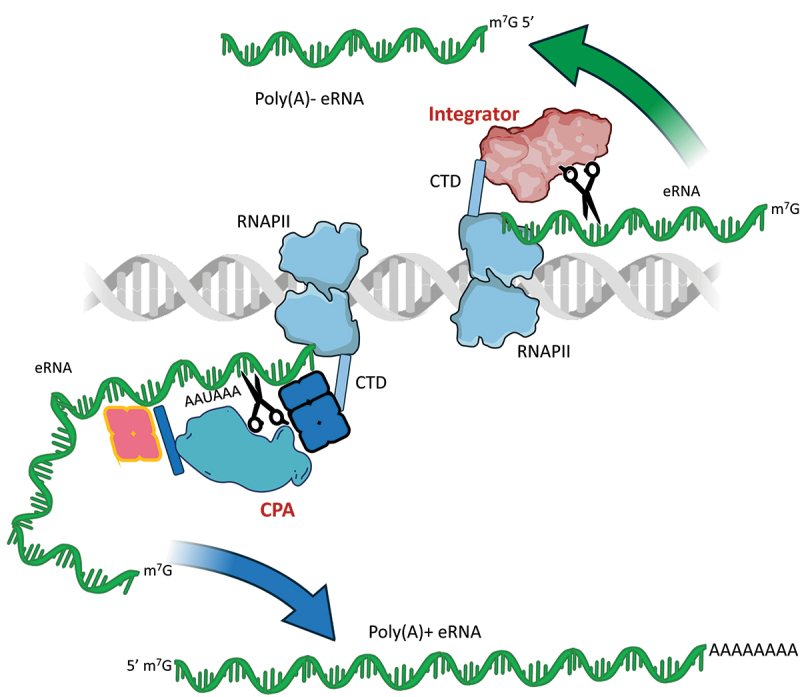
Termination pathways of eRNAs.

Taken together, the findings discussed above indicate the existence of multiple forms of the same eRNA transcripts produced by different processing machineries. While questions remain such as the prevalence of polyadenylation signals (PAS) in eRNA transcripts (Figure 2) and the extent of such eRNA variation across the wide range of transcribed enhancers in the human genome, these pathways do produce two distinct types of eRNAs, some Integrator-cleaved and non-polyadenylated and others polyadenylated, likely by the canonical polyadenylation (PA) machinery (Figure 2). Integrator processing of eRNAs also relies on the elongation factor PAF1C . [159]PAF1C facilitates recruitment of Integrator and depletion of the PAF1 subunit leads to accumulation of longer eRNA transcripts and enrichment of canonical PAS sequences in their 3’ regions [160]. PAF1C was also detected at active enhancer and SE sites and was found to positively regulate enhancer activity at these sites [161]. Additionally, a study linking enhancer activity to alternative PAS usage has proposed that enhancers recruit PA factors that then travel with RNAPII [162], a process that could facilitate eRNA polyadenylation. The presence of canonical PAS sequences in longer, more stable eRNAs is supported by the analysis of PAS sequencing data [17,163,164].

eRNAs can be terminated by either the Integrator (top) or the Cleavage and Polyadenylation (CPA) complex (bottom), similar to other RNAPII transcripts. Both of these complexes bind to the C-terminal domain (CTD) of RNAPII, but terminate eRNAs at different stages of transcription. Early in transcription, Integrator can cleave an eRNA to form a shorter, non-polyadenylated transcript that can then function in enhancer-associated transcription activation (top). Alternatively, eRNAs that are not cleaved by Integrator are longer and are instead terminated at a polyadenylation site (including the AAUAAA signal) by the CPA complex (bottom). These polyadenylated eRNAs are then either targeted for exosomal degradation by Mtr4 and the PAXT/PPC complex or exported from the nucleus.

**Figure 3.**
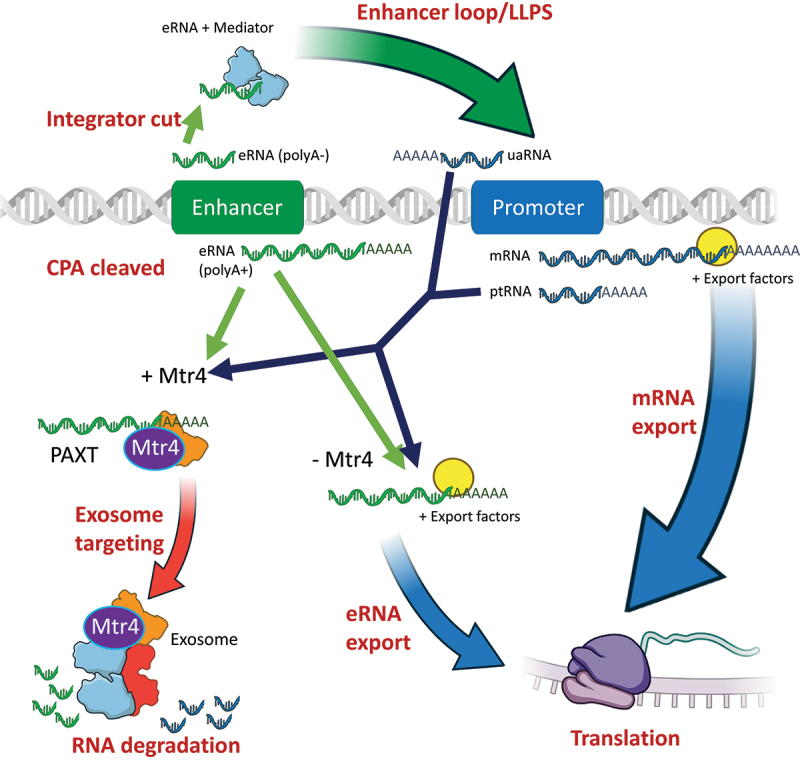
RNA surveillance and possible fates of eRNAs.

The above observations imply the possible existence of a branching pathway that determines eRNA fates (Figure 3). In such a model, the first ‘branch’ occurs with Integrator cleavage: eRNAs processed by the Integrator relatively early in their transcription are released from RNAPII and can be associated for example with Mediator and Cohesin to help form enhancer loop structures [10,155]. This type of Integrator processing is well-known in the generation of other small nuclear RNAs [165,166]. When the enhancer loop dissociates, these polyA- eRNAs can then be targeted for exosomal degradation by NEXT [74]. eRNAs that escape Integrator cleavage are transcribed farther and eventually polyadenylated, similar to their stabilization following depletion of Integrator recruitment factors [160]. These polyA+ transcripts then reach the second ‘branch’ where they are either targeted by PAXT/PPC for exosomal degradation [91] or exported to the cytoplasm, if they are instead stabilized and interact with export factors. This model is a variation of the previously discussed ‘redundant layer’ model of NEXT and PAXT surveillance [29,83,150], adding two more possible fates: early termination and Mediator association specific to eRNAs, and nuclear export followed by possible translation or other cytoplasmic functions. Notably, NEXT-targeted transcripts that avoid degradation can also be exported as part of an additional regulatory layer [83]. In the final eRNA fate option, nuclear export of polyA+ eRNAs, these transcripts blur the line between canonical protein-coding and -noncoding RNAs (Figure 3). While the existence of the individual ‘branches’ themselves is clear, the mechanism behind eRNA fate determination, i.e. which branch is selected, is not yet known. Considering the aforementioned connection of eRNAs and short vs long RNAs in general with transcriptional condensate formation and dissolution [141,143], the dynamics of these structures may play a role in this decision.

Differential eRNA processing can lead to distinct fates for these transcripts. Cleavage by the Integrator complex early in eRNA transcription can lead to interaction between polyA- eRNAs and the Mediator complex as part of the process of enhancer loop formation, or in some cases, e.g. super-enhances, LLPS. In the absence of Integrator cleavage, extended and polyadenylated eRNAs may be targeted by the Mtr4-containing PAXT complex for exosomal degradation. In the absence of both Integrator and PAXT interaction, eRNAs can instead interact with nuclear export factors and be exported to the cytoplasm, possibly for translation. These latter fates are similar to promoter-associated transcripts where mRNAs are exported for translation while uaRNAs and prematurely terminated (pt) RNAs are similarly targeted by PAXT or other Mtr4-containing complexes for exosomal degradation.

eRNAs and enhancer activation more generally have been studied extensively in the context of stem cells. Given the complex network of regulatory events required for maintenance of stem cell features and differentiation into distinct cell types, enhancers play major roles [116, 167–169]. Many enhancer sites involved in these processes are SEs, including those that establish cell fate, and exhibit high levels of eRNA transcription [115,117,118] and polyadenylation [121]. Furthermore, lncRNAs involved in stem cell function include transcripts associated with enhancer and SE sites, representing examples of (likely) eRNAs playing roles in cellular identity and differentiation [122,170,171]. In one recent example, an SE-associated eRNA was found to play important roles in regulation of *Nanog* transcription and maintenance of stem cell pluripotency, including evidence for enhancer looping in this interaction [172]. Other examples of SE-associated eRNAs have been linked to cancer development and autoimmune disease, both with implications for their role in early development as well [173].

## Translation of ‘noncoding’ RNAs

Over the past decade, translation outside of canonical mRNAs has been identified as a source of novel peptides/proteins and a previously unrealized function for many RNA species. The development of ribosome profiling has allowed for the detection of non-canonical translation events across the human genome [16]. Most studies in this emerging field have focused on small ORFs (dubbed sORFs or smORFs) less than 300 nt in length, considerably smaller than typical protein-coding genes, though, as discussed below, larger ORFs are being increasingly detected. These sORFs have been identified both in canonically noncoding RNAs as well as in the 5’ and 3’ untranslated regions (UTRs) of annotated mRNAs and the mechanisms and functional significance behind both their translation and their interactions with canonical mRNA ORFs, both in normal cells and in disease, have been the subject of many recent studies (e.g. [174–177], reviewed by [13,178–180]).

One major area where ‘noncoding’ RNAs have been found to be translated and produce functional peptides is in cancer. As mentioned previously, lncRNAs have been connected to many different areas of cancer development and diagnosis, and the peptide/protein products encoded by some have received increased attention. Examples of such lncRNA-encoded peptides range across many different cancer types, and include both smaller peptides and proteins, defined as >100 aa [181–183]. In the case of the proteins, examples include a 130 aa protein that enhances stem cell-like properties of breast cancer by activating the wnt/β-catenin pathway [184] and a 189 aa protein encoded by a cancer-associated lncRNA splice variant involved in enhancing proliferation in hepatocellular cancer by stabilizing nucleolin and increasing rRNA transcription [185]. Other recently discovered examples of short peptides, both from hepatocellular carcinoma, include a 60 aa peptide that associates with the tumour suppressor RBM10 and promote the epithelial-mesenchymal transition [186], while another peptide of 70 aa promotes proliferation and invasion by activating the MAPK/ERK pathway [187]. Other lncRNA-encoded peptides in contrast play roles in tumour suppression, either directly or as targets for the anti-tumour immune response. Notable examples include a 60 aa lncRNA-encoded peptide involved in suppressing angiogenesis in breast cancer by down-regulating STAT3 phosphorylation [188], while a group of multiple short peptides that are presented on cancer cell surface MHC class I complexes are immunogenic and can drive a CD8 T lymphocyte response resulting in cell death [189]. All of these are considered promising targets for novel anti-cancer therapies, whether through direct suppression of tumour development or as a ‘cancer vaccine’ in the case of the MHC class I associated peptides. What distinguishes these ‘coding’ lncRNAs from canonical mRNAs is that they were all shown to have typical lncRNA-related functions before their mRNA-like functions were uncovered.

LncRNA-encoded peptides/proteins are not exclusive to cancer-related pathways, however, and there is growing evidence for their wider role in normal cellular function. In one such example, a small histone-binding peptide encoded by a lncRNA was found to act as a transcriptional regulator for a wide range of genes [12]. Another lncRNA-encoded peptide was found to play a role in cell movement during the gastrulation stage of early embryonic development [190]. Protein-coding sequences have even been found in lncRNAs with their own well-studied and established regulatory functions. For example, a peptide encoded in the 5’ region of MALAT1, a typically nuclear lncRNA implicated in multiple aspects of gene expression, plays a role in neuronal function, and other, as yet unstudied, ORFs are present in the same region [191]. Another example is the lncRNA PAPAS, a nucleolar RNA transcribed antisense to the rRNA locus and involved in rRNA regulation [192,193]. PAPAS contains an ORF encoding an ~25 KDa protein, RIEP, found to play a role in the heat shock response in both the nucleolus and mitochondria [11]. Recently, another nucleolar-transcribed RNA downstream of RIEP, called ‘ORF3’, has been found to encode a ~ 22 kDa protein that exhibits RNA-mediated nuclear localization [194].

A fraction of eRNAs has also recently been found to be capable of encoding proteins. Specifically, over 15% of eRNAs identified in human cells were found to have ORFs over 300 nt long, encoding proteins up to almost 50 KDa [17]. These proteins were identified initially by ribosome profiling of cells depleted of Mtr4 (to increase eRNA abundance), and many were subsequently found to accumulate in a variety of cell types based on analysis of MS proteomic databases. While Mtr4 knockdown was used in these experiments to stabilize eRNAs and thereby increase the likelihood of detecting translated eRNAs, it is notable that Mtr4 levels naturally vary between different cell types [92–94], raising the possibility that levels of Mtr4 target transcripts, and thus possible encoded proteins, may vary in physiologically significant ways. Strikingly, as is also the case with REIP, the majority of the characterized eRNA-encoded proteins are very basic and arginine rich, primate-specific, and many localize to the nucleus [17]. These shared traits have implications for both a common set of cellular functions and the possibility that these ORFs are examples of de novo gene birth, as discussed below.

## Basic proteins and arginine richness

RIEP and most of the characterized eRNA-encoded proteins are highly basic (pI > 11) and contain a high arginine to lysine ratio, which is unusual among human proteins. Although possible functions for eRNA-encoded proteins have yet to be established, one function typically associated with arginine-richness is interaction with nucleic acids, both DNA and RNA, which is the case for ribosomal proteins [195], histone proteins and many splicing factors [196]. Similarly, protamines, small histone-like proteins expressed in sperm cells, are extremely arginine-rich and lack lysines [197,198]. This arginine-richness appears to be the result of evolutionary selection for greater DNA compaction, thereby increasing sperm competitiveness [199,200]. This view is supported by the differences in physical and chemical interactions between DNA and lysine- vs arginine-rich proteins: arginine, due to its guanidinium group, leads to tighter and more thermostable interactions between proteins and nucleic acids and therefore tighter packing of DNA [199,201,202].

Another notable area of protein function affected by arginine-richness is LLPS. As mentioned earlier, LLPS is the process by which biomolecular condensates within the cell form membraneless organelles with distinct biological functions [203]. Arginine-rich proteins that interact with RNAs are involved in the formation of Cajal bodies in the nucleus, stress granules in the cytoplasm, and other similar structures [204,205,206]. As with stronger protein-nucleic acid interaction, the chemical differences of arginine and lysine play a role here as well: greater hydrophobicity, lower solubility, and stronger cation-π interactions with aromatic groups all contribute to stronger LLPS behaviour [207]. It is notable in this regard that certain enhancers, especially SEs, are thought to undergo LLPS through the formation of transcriptional condensates [6,145], and eRNAs are likely to play a role in this process [142,143,208]. A speculative possibility is that eRNA-encoded proteins do as well, a possibility supported by their arginine-richness, nuclear localization and predicted disordered structure [17]. Additionally, mRNAs encoding arginine-rich, condensation-prone proteins have recently been found to undergo additional layers of regulation, likely to prevent accumulation of proteins that can be toxic at high doses [209]. The ‘branching paths’ of eRNA processing described above could have a similar effect in modulating accumulation of eRNA-encoded proteins.

The arginine-rich nature of eRNA-encoded proteins is likely linked to the GC-rich nature of their loci, as four of the six arginine codons are CGN. The presence of CpG islands at enhancer sites is well documented, especially in the case of orphan CpG islands located far from canonical promoters. In particular, CpG islands are associated with highly active enhancers and play a role in regulating their interaction with target genes [210–212]. eRNA transcription has also been associated with higher enhancer activity, including that of clustered SEs [100,129]. GC-richness is also a feature of other lncRNA loci, such as the rRNA locus encoding RIEP [11,213]. Interestingly, CGN bias in arginine codons has been linked with both higher rates of de novo mutations and with essential genes, both of which are often associated with genetic disease risk [214]. Despite their disease association, such high mutation rates suggest that CpG-rich transcripts, including eRNAs, have the potential to serve as sources of novel proteins for de novo gene birth and for evolution to act on, as discussed in the following section.

## eRNAs and De novo gene evolution

**Figure 4.**
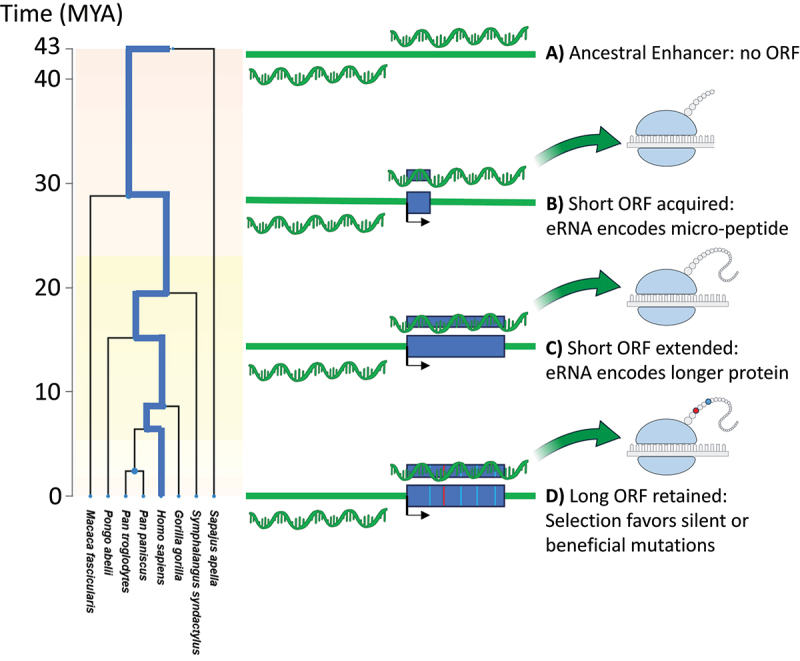
De Novo gene birth and evolution at enhancer sites.

Characterized eRNA ORFs display several properties that suggest a possible role as sources of novel genes. For example, as mentioned above, enhancers exhibit higher mutation rates than canonical protein-coding genes [20,21], a property that has mostly been associated with the rapid evolution of their regulatory function and transcription factor binding [215]. Enhancers rich in orphan CpG islands have also been linked to rapid evolution and the recent acquisition of function [19]. However, high mutation rates have the potential to play another role, specifically in producing novel proteins for evolution to act on, thereby leading to the creation of new functional genes. Novel species-specific genes are thought to arise in several ways, including by the process known as de novo gene birth [18,34]. In the case of eRNAs, we propose a model for their generation that parallels that of ‘transcript-first’ de novo gene evolution. In this model, an existing transcript, i.e. the eRNA, acquires a short ORF that either undergoes non-functional translation or produces neutral micro-peptides [216], which can then evolve to gain a function that can in turn be subject to stabilizing selection [18,33,217,218], see Figure 4. Additionally, computer models of this process have predicted that such ORFs begin as relatively short sequences and are subsequently either extended, leading to their establishment as novel genes, or lost through additional mutations [219,220]. Notably, in general, novel ORFs tend to be G/C-rich [221,222], analogous to eRNA-encoded ORFs.

This model for generation and evolution of eRNA ORFs is based on the ‘transcript first’ model of de novo gene birth, where ORFs are gained through mutations in transcribed regions, and are then subject to further mutation and selection [18,33]. The model presented depicts the evolution of the human version of the ORF (left, blue line), and other lineages in the tree may have other sequences derived from a common ancestor, either lacking or losing an ORF or developing their own. A) The ancestral version of the enhancer begins with no ORF sequence, but with eRNAs transcribed bidirectionally through established enhancer activation mechanisms. B) In the next step, a small ORF forms as a result of random mutations in the enhancer sequence, likely producing a micro-peptide if translated. Such translation may be non-functional at this stage, or give rise to a functional micro-peptide after further mutations. C) Over time, mutations cause the small ORF to be extended, creating a sequence that encodes a larger peptide/protein, which is both more stable and subject to further evolutionary selection. D) As the larger ORF is retained, selection favours either neutral or beneficial mutations, leading to the eRNA site becoming an ‘established’ gene with a functional protein product.

Novel genes, including those perhaps arising from some eRNAs, can be species-specific. For example, computational studies have confirmed the presence of species-specific intergenic ORFs near enhancer sites, such as in mice, predicted to be de novo genes either absent or lacking protein-coding function in more distant species [22]. More generally, species-specific ORFs, also called orphan genes, are a well-known phenomenon contributing to the genomic differences between closely-related species, including humans and other primates [223–225]. Additionally, studies of predicted structure and function across de novo genes in *Drosophila* have identified novel proteins predicted to form LLPS condensates [24]. Orphan genes are not exclusive to canonical coding sequences, as human-specific proto-genes and functional lncRNAs have also been observed [226–228]. Human-specific transcripts have been considered as a source of novel functional peptides [23], with recent studies linking novel human-specific proteins to ORFs encoded by lncRNAs [229,230]. In another interesting example, a human-specific ORF encoding a predicted 107 aa protein with a transmembrane domain was identified in *SMIM45*, a gene containing a much more widely conserved 68 aa ORF, likely with lncRNA ancestors identified in even more distantly related species [231].

Recent advances in single-cell RNA-seq and proteomics have provided further insight into the expression and function of de novo genes and their encoded proteins. Notably, two tissues strongly associated with novel gene expression are the brain and testis, where this phenomenon has been observed at both the RNA [232] and protein [233,234] levels. Extensive RNA-seq and proteomic analyses of novel genes have been performed in model organisms such as *Drosophila*, and expression patterns match those in humans, i.e. in the brain and testis [235], along with other regions such as the immune system [236]. The presence of human-specific genes in the brain is not surprising, especially given the human-specific expansion and folding of the cerebral cortex (reviewed by [237]). Such genes are present across different cell types in the brain, including glia and microglia, and have been linked with foetal brain development and differentiation of neuronal cell types [238–242]. Similarly, expression of de novo genes and their protein products has been detected in blood and the differentiated cell types present therein, including immune cells [236]. This evolution is also capable of producing human-specific functional lncRNAs, such as one found to play a role in GATA2 and HBG activation during erythropoiesis through chromatin modification [229]. The presence of novel genes in the testis and related to sperm function [236,243] is also connected to the ‘testis-first’ model of de novo gene birth, in which the testis is a major site of novel mutations and genes, contributing both to improved sperm function and other heritable traits (reviewed in [34]). It is also notable, as mentioned above, that genes encoding the two protamines display many of these properties. Furthermore, proteomics data on eRNA protein interactomes suggest possible connections to both sperm and immune function [17].

## Conclusions and future directions

Pervasive transcription of the human genome, the regulation and function of its RNA products, and the various pathways involved have been a major part of the study of RNA biology. This regulation occurs at multiple stages of transcription and includes a network of different complexes with distinct roles and targets [29,70]. eRNAs constitute an important example of this regulatory complexity, as these transcripts are both similar to and interact with transcripts from canonical promoters and can also represent an ambiguity between coding and noncoding RNAs. This ambiguity is further highlighted by the possibility that both polyadenylated and non-polyadenylated transcripts are produced not only from enhancers (eRNAs, Figure 2) but also from some promoters (both lncRNAs and mRNAs [125,244]. These similarities between RNAs produced from enhancers and promoters extend the functional similarities between the two elements, e.g. that enhancer activity can be attributed to some promoters [2]. In the case of eRNAs, and lncRNAs more generally, the branching pathways we proposed (Figure 3) may be beneficial in producing multiple RNAs with distinct functions from the same enhancer/promoter site through modulation of different regulatory complexes: short vs long and polyA+ vs polyA- RNAs can fulfill different roles, with varying degrees of ‘mRNA-likeness’ or ‘lncRNA-likeness’. Furthermore, branching pathways may allow for the evolution of novel functional RNAs, whether coding or noncoding, through mutations that affect one variant but not another or which alter the balance of different RNA products made at these sites. New elements of this regulatory network are still being discovered [82,83,163], and it is likely that further studies will provide additional insights into the multifunctional nature of both ‘coding’ and ‘non-coding’ RNAPII transcripts.

We have highlighted in this review the possibility that ‘lnc’ RNAs, including eRNAs, provide a ‘hidden proteome’ of novel proteins. This hidden proteome hypothesis [245] has been investigated more broadly by projects such as OpenProt, which have linked existing MS data with previously unannotated ORFs [246]. Indeed, both human-specific lncRNAs and lncRNA-encoded peptides/proteins have been proposed to be the products of de novo gene evolution [229,230,247]. Human-specific and primate-specific ORF status and novel functions have also been linked to new regulatory roles in a variety of cells and tissues [248–250].

Enhancers themselves also offer the potential to play important roles in the process of de novo gene birth. Indeed, the vast number of enhancers, 300,000 or more in humans [130,131], suggests a correspondingly immense number of transcripts (eRNAs) as starting material for the generation of new genes. Additionally, the typically CpG-rich nature of enhancers not only potentially makes them a source of arginine-rich proteins, possibly involved in regulatory functions through interactions involving nucleic acids and/or RNA/DNA binding proteins [199,202], but also drives higher mutation frequency, an important facilitator of de novo gene evolution [20,214]. It is notable that the wide range of enhancer activation states across diverse cell types implies a corresponding wide range of cell-specific eRNA expression patterns and thus a varied cellular milieu in which novel proteins might evolve. Altogether, the possibility that enhancers serve as a source for de novo gene birth not only adds a new layer to the important roles enhancers play in determining cell fate but also extends significantly the reservoir of transcripts available to serve as the ‘raw material’ for the evolution of new genes. Future studies investigating these and related topics promise to be exciting.

## Data Availability

This is a review article. Data sharing is not applicable to this article as no data were created or analysed in this study.
